# Exosomal EPHA2 derived from highly metastatic breast cancer cells promotes angiogenesis by activating the AMPK signaling pathway through Ephrin A1-EPHA2 forward signaling

**DOI:** 10.7150/thno.72404

**Published:** 2022-05-13

**Authors:** Baoai Han, He Zhang, Ruinan Tian, Hui Liu, Zhaosong Wang, Zhiyong Wang, Jianfei Tian, Yanfen Cui, Sixin Ren, Xiaoyan Zuo, Ran Tian, Ruifang Niu, Fei Zhang

**Affiliations:** 1 Public Laboratory, Tianjin Medical University Cancer Institute and Hospital, National Clinical Research Center for Cancer, Tianjin, 300060, China.; 2 Key Laboratory of Cancer Prevention and Therapy, Tianjin, 300060, China.; 3 Tianjin's Clinical Research Center for Cancer, Tianjin, 300060, China.; 4 Key Laboratory of Breast Cancer Prevention and Therapy, Ministry of Education, Tianjin, 300060, China.

**Keywords:** exosomes, angiogenesis, high metastatic potential cells, breast cancer, EPHA2

## Abstract

**Rationale:** Angiogenesis is a fundamental process of tumorigenesis, growth, invasion and metastatic spread. Extracellular vesicles, especially exosomes, released by primary tumors promote angiogenesis and cancer progression. However, the mechanism underlying the pro-angiogenic potency of cancer cell-derived exosomes remains poorly understood.

**Methods:** Exosomes were isolated from breast cancer cells with high metastatic potential (HM) and low metastatic potential (LM). The pro-angiogenic effects of these exosomes were evaluated by in vitro tube formation assays, wound healing assays, rat arterial ring budding assays and in vivo Matrigel plug assays. Subsequently, RNA sequencing, shRNA-mediated gene knockdown, overexpression of different EPHA2 mutants, and small-molecule inhibitors were used to analyze the angiogenesis-promoting effect of exosomal EPHA2 and its potential downstream mechanism. Finally, xenograft tumor models were established using tumor cells expressing different levels of EPHA2 to mimic the secretion of exosomes by tumor cells in vivo, and the metastasis of cancer cells were monitored using the IVIS Spectrum imaging system and Computed Tomography.

**Results:** Herein, we demonstrated that exosomes produced by HM breast cancer cells can promote angiogenesis and metastasis. EPHA2 was rich in HM-derived exosomes and conferred the pro-angiogenic effect. Exosomal EPHA2 can be transferred from HM breast cancer cells to endothelial cells. Moreover, it can stimulate the migration and tube-forming abilities of endothelial cells in vitro and promote angiogenesis and tumor metastasis in vivo. Mechanistically, exosomal EPHA2 activates the AMPK signaling via the ligand Ephrin A1-dependent canonical forward signaling pathway. Moreover, inhibition of the AMPK signaling impairs exosomal EPHA2-mediated pro-angiogenic effects.

**Conclusion:** Our findings identify a novel mechanism of exosomal EPHA2-mediated intercellular communication from breast cancer cells to endothelial cells in the tumor microenvironment to provoke angiogenesis and metastasis. Targeting the exosomal EPHA2-AMPK signaling may serve as a potential strategy for breast cancer therapy.

## Introduction

Breast cancer remains a major threat to women worldwide due to its relatively high morbidity and mortality 1-3. The occurrence of metastasis is the leading cause of treatment failure and death in patients and a major challenge in the clinical management of breast cancer 4, 5. Cancer metastasis is extremely complex; it not only requires tumor cells to evolve stronger invasive and metastatic capabilities but also requires them to induce a favorable microenvironment to facilitate tumor cell colonization and growth. Accumulated evidence suggests that the metastatic niche is required for tumor cells to metastasize from primary sites to distal regions. The metastatic niche is characterized by angiogenesis, lymphangiogenesis, inflammation, vascular permeability, immunosuppression, organ repulsion and reprogramming. Angiogenesis is critical for establishing a metastatic microenvironment and subsequently promoting tumor cell extravasation, intravasation, survival, proliferation and metastatic outgrowth 6-8. However, the underlying mechanisms regulating this process during metastatic niche formation remain poorly understood and therefore need to be elucidated.

The metastatic microenvironment consists of extracellular matrix proteins, inflammatory immune cells, endothelial cells, fibroblasts and tumor cells with heterogeneous populations 9, 10. The crosstalk between cancer cells and stromal cells in the tumor microenvironment regulates angiogenesis and promotes cancer progression 11. Recent studies have revealed that extracellular vehicles, especially exosomes, mediate intercellular communications by carrying proteins, peptides, nucleic acids and lipids. For instance, the exosome-mediated transfer of miR-210 from fibroblasts to endothelial cells promotes angiogenesis and tumor growth in lung cancer 12; exosomes derived from tumor-associated macrophages promote angiogenesis and cervical cancer progression by delivering TIE2 13; exosomal HMGB1 derived from hypoxia-conditioned bone marrow mesenchymal stem cells enhances angiogenesis through the JNK/HIF-1α signaling pathway 14. Moreover, tumor cells secrete exosomes and contribute to angiogenesis and the formation of the metastatic niche. Cancer cells within a tumor are a highly heterogeneous population with different proliferative, drug-resistant and metastatic capabilities. A recent study showed that drug-resistant colon cancer cells promote angiogenesis through exosomal DPP4 15; two recent studies indicated that intratumor communications between high-metastatic and low-metastatic cancer cells enhance metastasis 16, 17. However, whether these heterogeneous cancer cells differ in promoting angiogenesis is unclear. In this study, we demonstrated that EPH receptor A2 (EPHA2) is enriched in exosomes derived from breast cancer cells with high metastatic potential (HM) and promotes angiogenesis and metastasis.

EPHA2 is a member of the EPH receptor tyrosine kinase family. In normal tissues, EPHA2 is expressed on the membrane of epithelial cells and interacts with Ephrin A1, its ligand that is expressed on the surface of adjacent cells 18, 19. The binding of Ephrin A1 to EPHA2 occurs in cell-cell contacts and induces bidirectional signals in the corresponding cells, forming a critical cell-cell communication system 20. The forward signaling pathway is transmitted from Ephrin A1 to EPHA2 and is usually accompanied by the activation of EPHA2 kinase, which is characterized by Tyr588 phosphorylation (canonical signaling). In contrast, the signal transmitted from EPHA2 to Ephrin A1 is known as the reverse signaling pathway and is independent of the kinase activity of EPHA2. Besides, EPHA2 also mediates ligand-independent forward signaling, which is characterized by phosphorylation at S897 (noncanonical signaling) 21. The function of EPHA2 in cancer is complex and the EPHA2-mediated signaling system is always dysregulated in many tumors. In general, most studies support an oncogenic role for EPHA2 non-canonical signaling and a tumor suppressor role for canonical signaling 19. More interestingly, recent studies have found that EPHA2 can be expressed on exosomes, implying that EPHA2 can function independently of cell-to-cell contact, thus complicating EPHA2-mediated signaling 20, 22.

The upregulation of EPHA2 is associated with poor prognosis, increased metastatic potential and reduced survival in breast cancer patients 23-26. High expression of EPHA2 is an indicator of high tumor cell aggressiveness. Several studies suggest that EPHA2 has the potential to be an attractive target for cancer therapy. Nevertheless, the functional significance of the exosomal EPHA2 in breast cancer remains unclear. Therefore, the role and mechanism of exosomal EPHA2 in regulating breast cancer angiogenesis and metastasis need to be identified. In this study, we found that the exosomes produced by HM breast cancer cells were rich in EPHA2. The exosomal EPHA2 can be transferred from HM breast cancer cells to endothelial cells. Moreover, the exosome-transmitted EPHA2 promotes angiogenesis through canonical forward signaling in endothelial cells. Furthermore, AMPK signaling is downstream of the EPHA2 forward signaling pathway, and its activation confers and enhances the angiogenic ability of endothelial cells. Collectively, we demonstrated that HM breast cancer cells enhance their metastatic ability by promoting angiogenesis through exosomal EPHA2.

## Results

### Exosomes derived from HM breast cancer cells promote angiogenesis

**Figure 1:**
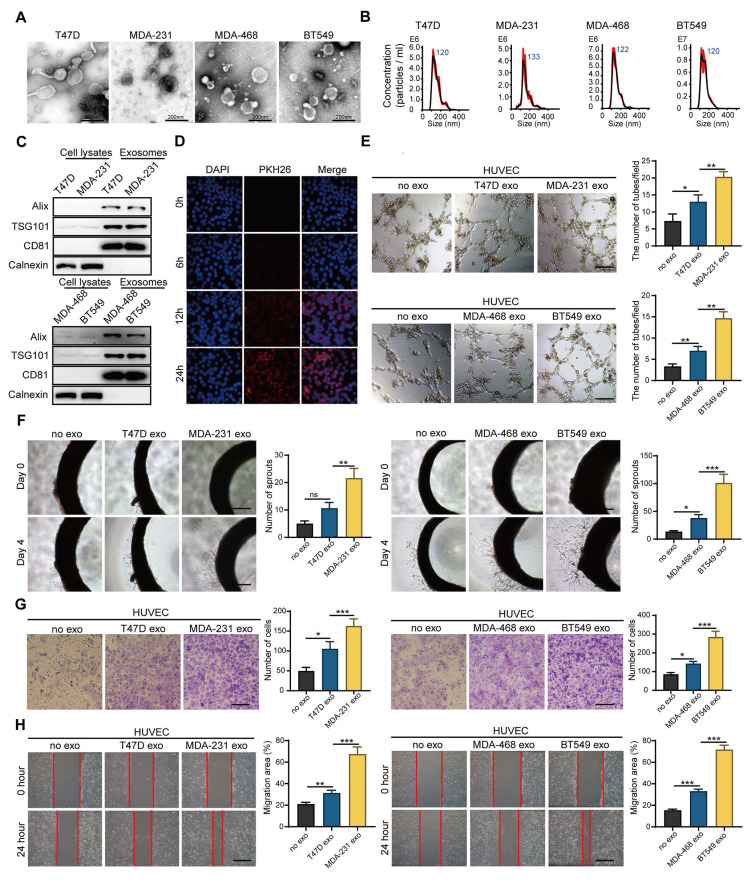
** Exosomes derived from breast cancer cells with high metastatic potential promote angiogenesis. A.** Transmission electron microscopy showed the morphology of exosomes derived from high or low-metastatic breast cancer cells. Scale bar: 200 nm. **B** Nanoparticle tracking analysis (NTA) of exosomes derived from high or low-metastatic breast cancer cells. The results showed that exosomes derived from 4 different breast cancer cells were comparable in size. **C** Western blot analysis of the isolated exosomes derived from indicated cell lines. Equal amounts of protein (100 μg) was loaded and analyzed. Alix, TSG101 and CD81 are specific markers for exosomes, while Calnexin was used to monitor cytoplasm contamination of isolated exosomes. **D** PKH-26-labeled exosomes can be endocytosed into the recipient cells. Scale bar: 200 μm. **E** HM-Exos significantly enhanced the tube-forming ability of endothelial cells compared to LM-Exos and control. Scale bar: 200 μm. **F** Compared with LM-Exos and control, HM-Exos significantly enhanced the capillary sprouting ability of rat arterial rings. Scale bar: 200 μm. **G, H** HM-Exos significantly enhanced the migration ability of endothelial cells compared to LM-Exos and control. Scale bar: 200 μm. Data are expressed as mean ± SD, and all experiments were repeated at least three times. *P < 0.05, **P < 0.01, ***P < 0.001 and ns P > 0.05 indicate no statistical significance. High Metastatic, HM; Ligh Metastatic, LM; Exosomes, Exos.

Tumors are a heterogeneous population, including cancer cells with different metastasis, proliferation and drug resistance potential. Intercellular communication in the tumor microenvironment mediated by exosomes plays a key role in promoting angiogenesis and accelerating cancer progression. To investigate whether breast cancer cells with different metastatic potential have different abilities to promote angiogenesis, we isolated exosomes (Exos) from breast cancer cells with HM (HM-Exos, isolated from MDA-231 or BT-549) and breast cancer cells with low metastatic potential (LM-Exos, isolated from MDA-468 or T47D). Then, we confirmed the structural features of exosomes by TEM (Figure 1A and Figure S1A) and nanoparticle tracking analysis (NTA) (Figure 1B). The diameter of purified exosomes ranged from 30 nm to 200 nm, and the exosomes were rich in exosome-specific markers, such as TSG101 and CD81, but lacked Calnexin, indicating that the exosomes were not contaminated by the cytoplasm (Figure 1C). Next, endothelial cells were incubated with PKH26-labeled exosomes. Figure 1D shows that the labeled exosomes could be internalized into the recipient cells. Afterward, the exosomes were further applied to endothelial cells, and the results showed that HM-Exos significantly enhanced the tube-forming ability of endothelial cells compared with LM-Exos (Figure 1E). Moreover, rat arterial ring assay revealed that HM-Exos exhibits a stronger ability to promote the outgrowth of arterial rings than LM-Exos, indicating that HM-Exos enhances vasculature generation (Figure 1F). In addition, HM-Exos induced a significant increase in the migration ability of endothelial cells compared with LM-Exos as measured by Transwell and Wound healing assays (Figure 1G-H). Altogether, these results demonstrated that HM breast cancer cells can endow endothelial cells with stronger capabilities, such as tube formation and migration through exosomes to promote angiogenesis.

### Exosomal EPHA2 promotes endothelial cell angiogenesis

**Figure 2:**
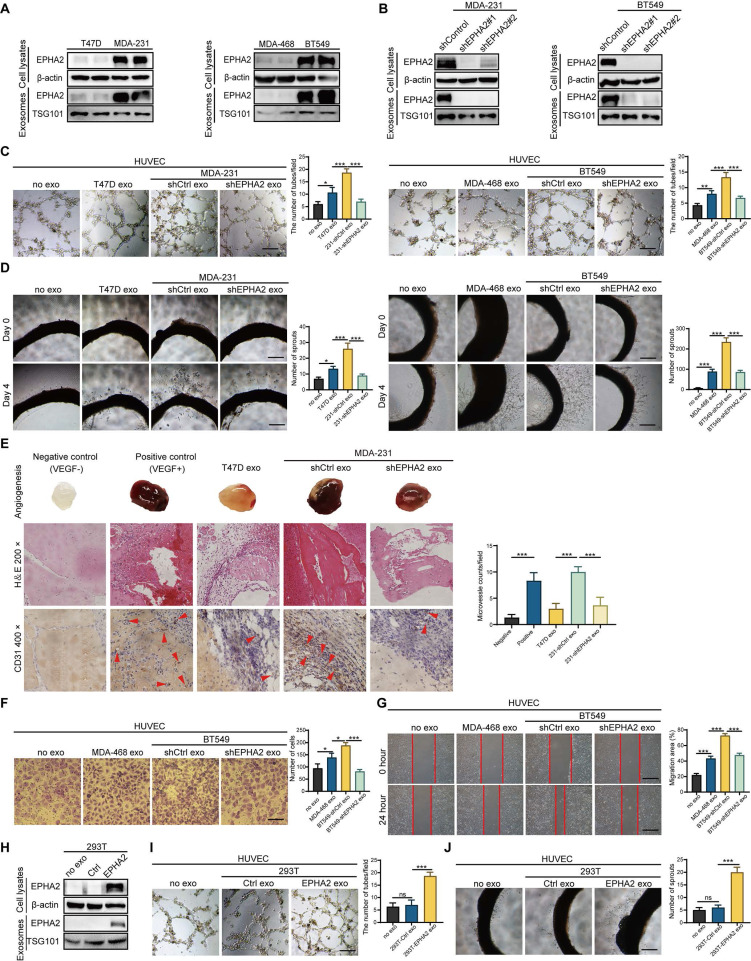
** Exosomal EPHA2 promotes endothelial cell angiogenesis. A** Expression of EPHA2 in exosomes and cell lysates was analyzed by using Western blotting; β-actin was used as the loading control. **B** Western blot analysis showed that EPHA2 expression was silenced in high metastatic potential cells and exosomes after infection with lentivirus expressing EPHA2-specific shRNAs; β-actin was used as the loading control. **C, D** EPHA2-silenced HM-exos failed to promote the tube formation of endothelial cells and the microvascular outgrowth of rat arterial rings. **E** Matrigel plug assay showed that exosomal EPHA2 promotes angiogenesis in vivo. For the in vivo matrix gel plug assay, Matrigel plugs with or without VEGF were used as positive or negative controls. HE and CD31 staining were carried out on the dissected Matrigel plugs. **F, G** EPHA2-silenced HM-exos failed to promote the migration of endothelial cells as measured by Transwell and Wound healing assays. **H** Western blot analysis showed the expression of EPHA2 in HEK-293T cells and exosomes in cells that were transfected with control or EPHA2 expression vectors. β-actin was used as the loading control. **I, J** Exosomes from HEK-293T cells expressing EPHA2 significantly promote the tube-forming capacity of endothelial cells compared with control exosomes. Data are expressed as mean ± SD, and all experiments were repeated at least three times. *P < 0.05, **P < 0.01, ***P < 0.001 and ns P > 0.05 indicate no statistical significance. Scale bar: 200 μm.

We recently found that EPHA2 is enriched in drug-resistant breast cancer cells and their exosomes, and the exosomal EPHA2 is required for breast cancer cell metastasis 20. Given that the metastatic ability of resistant cells in our study is stronger than that of sensitive cells, we tested the expression levels of EPHA2 in breast cancer cell lines with different metastatic potential. The results showed that the expression of EPHA2 in cells with HM is higher than that in cells with low metastatic potential (Figure 2A). Subsequently, we examined the expression levels of EPHA2 in the exosomes produced by the above cells, and the results showed that the expression of exosomal EPHA2 was consistent with that in total cellular proteins (Figure 2A). Next, HM breast cancer cells with stable knockdown of EPHA2 were constructed (Figure 2B), and their exosomes were collected to treat endothelial cells. The results showed that the ability of HM-Exos to promote endothelial cell tube formation was significantly reduced after knockdown of EPHA2 (Figure 2C). Moreover, HM-Exos from EPHA2-silenced cells failed to promote the outgrowth of rat arterial ring compared with control exosomes (Figure 2D). To observe the changes of angiogenic indexes in vivo, Matrigel plug assay was carried out, and the dissected Matrigel plugs were stained with HE and CD31. The results showed that the plugs from the HM-Exos treated group had more blood vessels than those in the LM-Exos treated group, and the plugs from the EPHA2 knockdown exosomes treated group had significantly fewer blood vessels (Figure 2E). In addition, the exosomes from EPHA2 stable silenced HM cells failed to promote the migration ability of endothelial cells as measured by Transwell and wound healing assays (Figure 2F-G and Figure S1B). To further determine the role of exosomal EPHA2 in promoting angiogenesis, we constructed HEK-293T cells overexpressing EPHA2 and collected exosomes (Figure 2H). Compared with control exosomes, EPHA2-rich exosomes significantly promoted the tube formation of endothelial cells and also increased the outgrowth of rat arterial rings in vitro (Figure 2I-J). In conclusion, these results suggest that exosomal EPHA2 is a critical mediator for HM breast cancer cells to promote angiogenesis.

### Exosomal EPHA2 promotes endothelial cell angiogenesis via Ephrin A1-EPHA2 forward signaling

**Figure 3:**
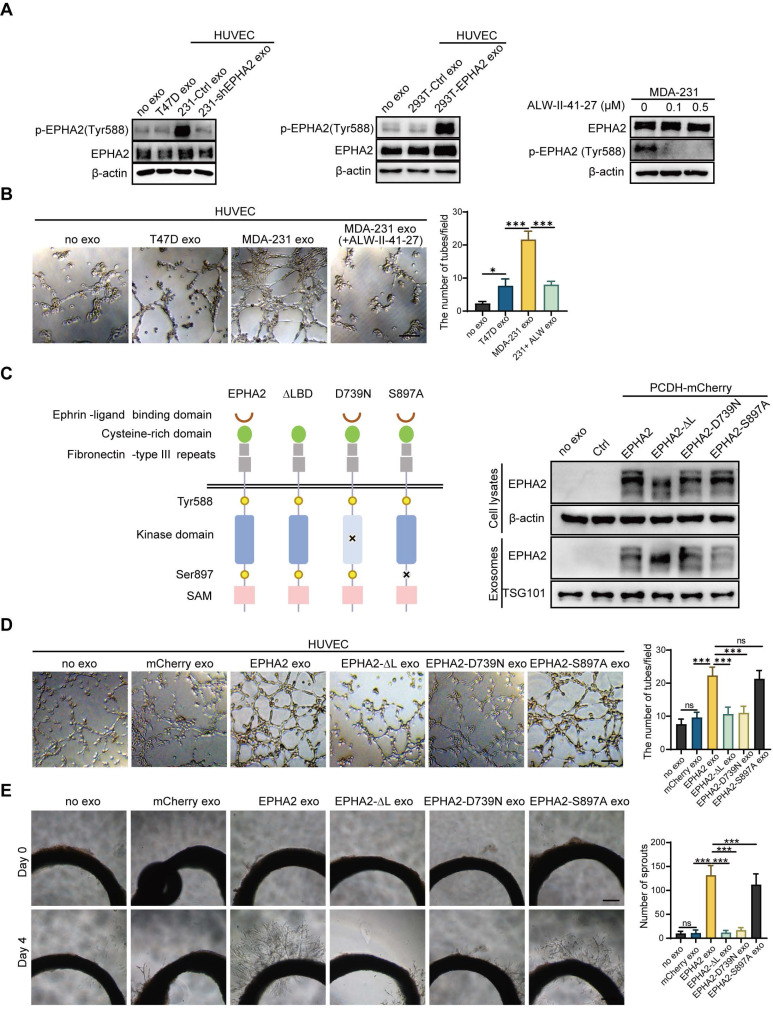
** Exosomal EPHA2 promotes endothelial cell angiogenesis via kinase-dependent forward signaling pathway. A** Uptake of HM-Exos by endothelial cells can enhance EPHA2 phosphorylation at Tyr588, whereas HM-Exos from EPHA2-silenced cells failed to induce an upregulation of EPHA2 Tyr588 phosphorylation (Left). EPHA2-rich exosomes from HEK-293T cells can induce a dramatic increase in EPHA2 Tyr588 phosphorylation in endothelial cells (Middle). HM breast cancer cells pre-treated with ALW-II-41-27, an inhibitor of EPHA2, can inhibit EPHA2 phosphorylation at Tyr588 when the cells were treated with HM-Exos (Right). **B** ALW-II-41-27 treated HM-Exos could not promote the tube formation of endothelial cells. **C** Schematic diagram of the structure of EPHA2 and its mutants. Full-length EPHA2 and its three mutants EPHA2-ΔL, EPHA2-D739N and EPHA2-S897A were cloned into pCDH-mCherry vector (Left). The expression of EPHA2 and its mutants in cell lysates and exosomes was detected by Western blotting. β-actin was used as a loading control (Right). **D, E** Exosomes carrying EPHA2 and EPHA2-S897A, but not EPHA2-ΔL and EPHA2-D739N, promote the tube-forming ability of endothelial cells and the sprouting capacity of arterial rings. Data are expressed as mean ± SD, and all experiments were repeated at least three times. *P < 0.05, **P < 0.01, ***P < 0.001 and ns P > 0.05 indicate no statistical significance. Scale bar: 200 μm.

Unlike conventional receptor tyrosine kinases, the interaction of EPHA2 and its ligand, Ephrin A1, can generate bidirectional signals in a kinase-dependent or independent manner. To investigate whether the kinase activity of EPHA2 is required for HM-Exos to promote angiogenesis, we treated endothelial cells with exosomes derived from LM and HM breast cancer cells. We found that HM-Exos uptake by endothelial cells not only significantly increased EPHA2 protein levels, but also enhanced EPHA2 phosphorylation at Tyr588, indicating the activation of EPHA2 kinase (Figure 3A-Left). Conversely, Exos from EPHA2-silenced HM cells failed to induce an upregulation of EPHA2 Tyr588 phosphorylation (Figure 3A-Left).

**Figure 4:**
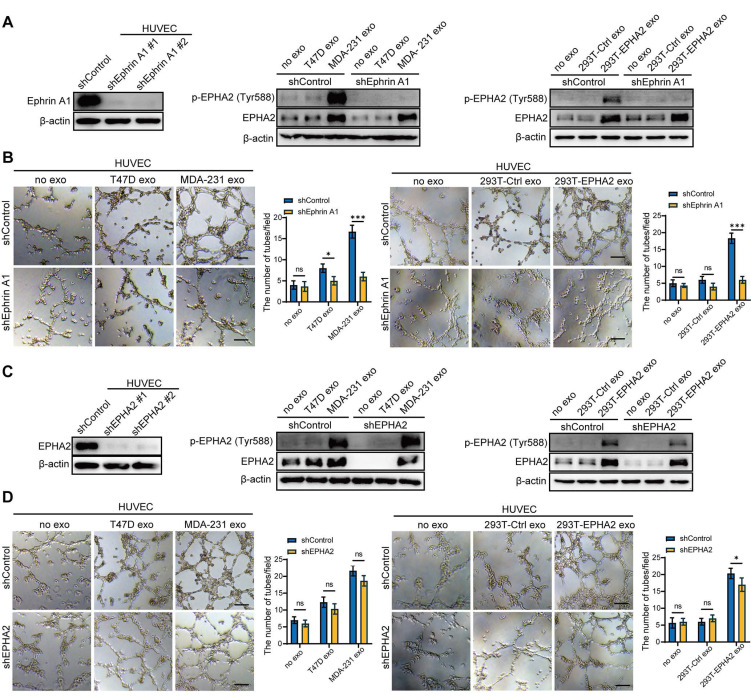
** Exogenous EPHA2 promotes endothelial cell angiogenesis through a ligand-dependent forward signaling. A** Western blot analysis of Ephrin A1 expression in lentivirus-infected HUVEC expressing control or Ephrin A1-specific shRNA (Left). HM-Exos failed to induce an upregulation of Tyr588 in Ephrin A1-KD HUVECs compared to control cells (Middle). Exosomes from EPHA2-expressing HEK-293T cells failed to induce an upregulation of Tyr588 in Ephrin A1-KD HUVECs compared with control cells (Right). **B** HM-Exos or EPHA2-rich exosomes failed to promote the tube-forming ability of Ephrin A1-KD cells. **C** Western blot analysis of EPHA2 expression in lentivirus-infected HUVEC expressing control or EPHA2-specific shRNA (Left). HM-Exos induces upregulation of Tyr588 in EPHA2-KD HUVECs compared to control cells (Middle). Exosomes from EPHA2-expressing HEK-293T cells induce an upregulation of Tyr588 in EPHA2-KD HUVECs compared with control cells (Right). **D** HM-Exos and EPHA2-rich exosomes still promote the tube-forming ability of EPHA2-KD HUVECs. All experiments were repeated at least three times. *P < 0.05, **P < 0.01,***P < 0.001 and ns P > 0.05 indicate no statistical significance. Scale bar: 200 μm.

Moreover, EPHA2-rich exosomes from HEK-293T cells induced a dramatic increase in EPHA2 Tyr588 phosphorylation in endothelial cells (Figure 3A-Middle). These results suggest that exosomal EPHA2 promotes endothelial cell tube formation in a kinase-dependent manner. To test this possibility, we treated HM breast cancer cells with ALW-II-41-27, an inhibitor of EPHA2 (Figure 3A-Right), and then isolated exosomes to treat endothelial cells. The results showed that the inhibitor-treated HM-Exos could not promote the tube formation of endothelial cells, supporting that the exosomal EPHA2 promotes endothelial cell tube formation dependent on its kinase activity (Figure 3B and Figure S1C). To further confirm our findings, full-length EPHA2 and its mutants EPHA2-ΔL, EPHA2-D739N and EPHA2-S897A were constructed into pCDH-mCherry and then transfected into HEK-293T cells. The expression of EPHA2 and its mutants in cells and exosomes was further determined (Figure 3C). As expected, exosomes carrying EPHA2-S897A still showed the ability to promote the tube formation of endothelial cells and the outgrowth of rat arterial ring in vitro, which was similar to the effect of exosomes carrying full-length EPHA2. However, exosomes carrying EPHA2-ΔL and EPHA2-D739N failed to promote endothelial cell tube formation and rat arterial ring outgrowth (Figure 3D, E). Therefore, these results suggest that the LBD domain and kinase activity are necessary for exosomal EPHA2 to promote angiogenesis. These data also indicate that exosomal EPHA2 promotes endothelial cell tube formation through a ligand- and kinase-dependent forward signaling pathway. To further confirm this hypothesis, we silenced Ephrin A1 expression in endothelial cells (Figure 4A-Left). As expected, HM-Exos and EPHA2-rich exosomes from HEK-293T cells failed to promote angiogenesis in Ephrin A1-KD cells (Figure 4B and Figure S1D). Moreover, these exosomes failed to increase the phosphorylation of EPHA2 at the Tyr588 site in Ephrin A1-KD cells (Figure 4A-Middle and Right). Collectively, these results suggest that exosomal EPHA2 promotes endothelial cell tube formation through the Ephrin A1-EPHA2 forward signaling pathway. HUVECs also express a certain level of endogenous EPHA2. To further distinguish whether endogenous EPHA2 is involved in angiogenesis induced by exosomes, we silenced EPHA2 expression in endothelial cells and treated them with HM-Exos or EPHA2-rich exosomes. The results showed that the phosphorylation of EPHA2 at the Tyr588 site in EPHA2-KD endothelial cells could be induced by these exosomes (Figure 4C). In addition, these exosomes induced enhanced angiogenesis in EPHA2-KD cells (Figure 4D and Figure S1E). This indicates that exogenous EPHA2 transmitted by exosomes is the main contributor to endothelial cell angiogenesis.

### Exosomal EPHA2 promotes endothelial cell angiogenesis through the AMPK signaling pathway

**Figure 5:**
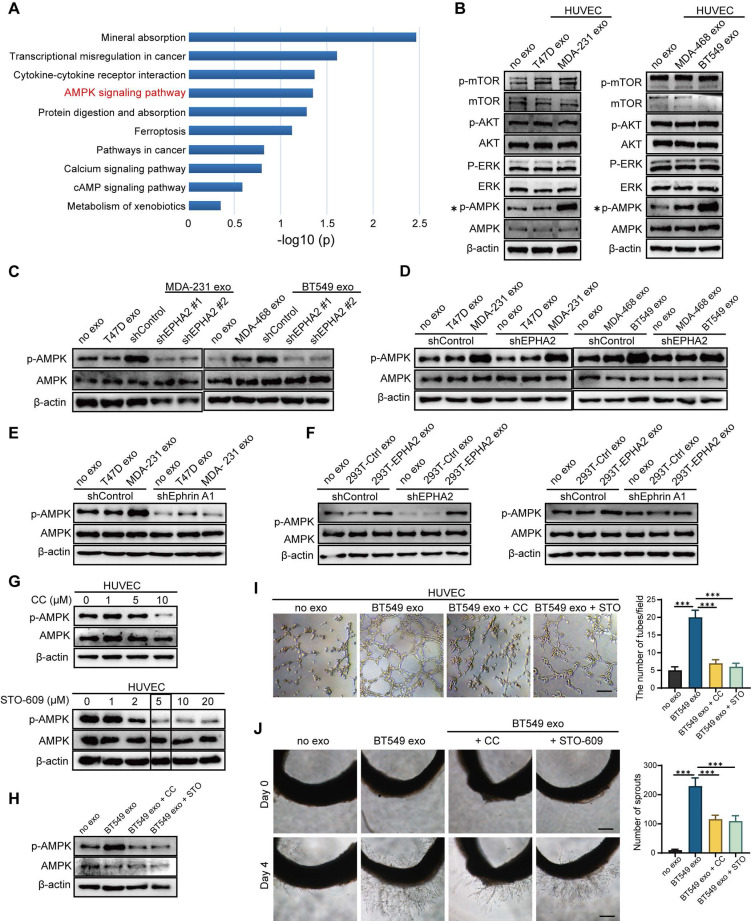
** Exosomal EPHA2 promotes endothelial cell angiogenesis through AMPK signaling pathway. A** KEGG enrichment analysis of the RNA-Seq data showed that the AMPK signaling pathway was significantly activated in EPHA2-rich exosomes treated HUVECs compared with control exosome treated cells. **B** The expression level of p-AMPK was significantly higher in the HM-Exos-treated endothelial cells than in the control and LM-Exos-treated cells, while the phosphorylation levels of Akt, mTOR and ERK were not altered. **C** Exosomes from EPHA2 stably silenced HM breast cancer cells failed to induce an upregulation of AMPK phosphorylation. **D** HM-Exos still induced an increase in AMPK phosphorylation in EPHA2-KD HUVECs compared with control cells. **E** HM-Exos failed to induce an upregulation of p-AMPK in Ephrin A1-KD HUVECs compared with control cells. **F** Compared with control cells, EPHA2-rich exosomes from HEK-293T cells could induce an increase in AMPK phosphorylation in EPHA2-KD HUVECs, but failed to induce an upregulation of phosphorylated AMPK in Ephrin A-KD HUVECs. **G** Compound C, an AMPK inhibitor, significantly inhibited AMPK phosphorylation at a concentration of 10 μM; STO609, a CaMKKβ inhibitor, significantly inhibited AMPK phosphorylation at a concentration of 5μM. **H** Compound C and STO609 eliminated phosphorylation of AMPK in HUVECs after incubation with HM-Exos. **I** Inhibition of AMPK signaling by Compound C and STO609 reduced tube-forming ability of HUVECs treated with HM-Exos. **J** Inhibition of AMPK signaling by Compound C and STO609 decreased the ability of microvascular outgrowth in HM-Exos-treated rat arterial rings. All experiments were repeated at least three times. *P < 0.05, **P < 0.01, ***P < 0.001 and ns P > 0.05 indicate no statistical significance. Scale bar: 200 μm.

To determine the mechanistic details through which exosomal EPHA2 induces angiogenesis, we treated HUVECs with EPHA2-rich exosomes and then analyzed the mRNA expression of endothelial cells by transcriptome sequencing (RNA-Seq). KEGG enrichment analysis showed that the activation of AMPK signaling pathway was significantly induced in EPHA2-rich-exosome-treated cells compared with control cells (Figure 5A ). In addition, GSEA analysis also showed induction of AMPK signaling pathway, but with a larger FDR value (Figure S2A). Consistently, the expression levels of phosphorylated AMPK (p-AMPK) were significantly higher in HM-Exos-treated endothelial cells than in control and LM-Exos-treated cells, whereas the phosphorylation levels of Akt, mTOR and ERK were not altered (Figure 5B and Figure S2B). In addition, HM-Exos from EPHA2 knockdown breast cancer cells failed to induce an upregulation of p-AMPK in endothelial cells (Figure 5C). These findings suggest that the AMPK signaling pathway is downstream of the Ephrin A1-EPHA2 forward signaling. Moreover, HM-Exos could still induce an apparent increase of p-AMPK in EPHA2-KD endothelial cells (Figure 5D). However, in Ephrin A1 knockdown endothelial cells, HM-Exos failed to induce an upregulation of p-AMPK compared with control cells (Figure 5E). Similarly, EPHA2-rich exosomes from HEK-293T cells can also induce an increase in AMPK phosphorylation in EPHA2-KD endothelial cells, but cannot promote the upregulation of p-AMPK in Ephrin A1-KD endothelial cells (Figure 5F). Therefore, these results suggest that the activation of AMPK signaling in endothelial cells induced by exosomes mainly depends on exogenous EPHA2 delivered by exosomes and the ligand Ephrin A1 expressed in endothelial cells. To further confirm the above findings, we pretreated endothelial cells with AMPK inhibitor Compound C and STO609, followed by HM-Exos or LM-Exos treatment. The results showed that Compound C and STO609 inhibited the upregulation of p-AMPK by HM-Exos (Figure 5G-H and Figure S3A). Moreover, HM-Exos failed to promote the tube formation of endothelial cells and the outgrowth of rat arterial rings in vitro in the presence of Compound C or STO609 compared with the control group (Figure 5I-J and Figure S3B-C). In addition, it has been shown that AMPK promotes angiogenesis by regulating the activity of HIF-1α 27. Herein, we found that HIF1-α silencing or its inhibition abolished the promoting effect of EPHA2-rich exosomes on the tube formation ability of HUVECs and the growth of rat arterial rings (Figure S4A-F). Taken together, these results suggest that breast cancer cell-derived exosomal EPHA2 promotes angiogenesis by activating the downstream AMPK- HIF1-α pathway through Ephrin A1-EPHA2 forward signaling.

### Exosomal EPHA2 promotes the metastasis of breast cancer cells in vivo

**Figure 6:**
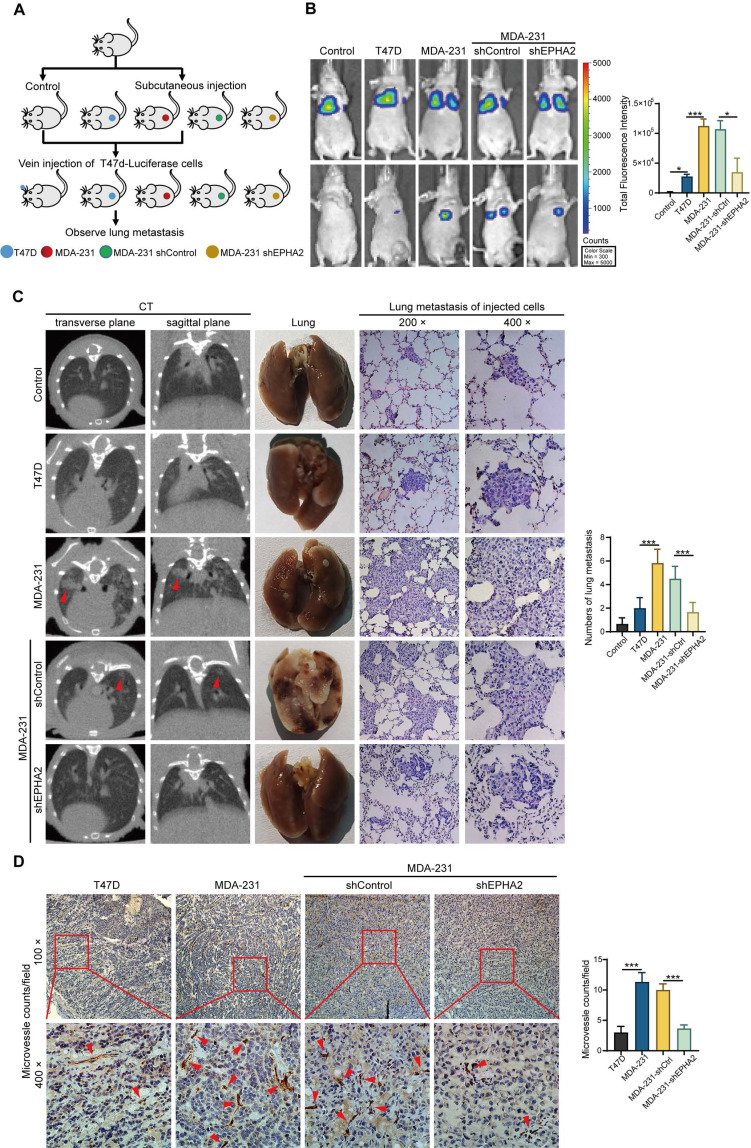
** Exosomal EPHA2 promotes the metastasis of breast cancer cells in vivo. A** Schematic diagram of the in vivo experiment. **B** Representative images showed the lung metastasis signals detected by IVIS Spectrum imaging systems. **C** The metastatic foci in the lungs of mice were investigated using Computed Tomography (CT) (Left). The number of metastatic foci on the lung surface was significantly reduced in the EPHA2 knockdown group compared with the control group (Middle). H & E staining showed that the number of micro-metastases in lung tissue of mice inoculated with HM breast cancer cells was significantly higher than that in the group inoculated with LM breast cancer cells (Right). **D** The IHC analysis showed that the microvessel density (MVD) in MDA-231 and MDA-231-shControl tumors was significantly increased compared with the T47D and MDA-231-shEPHA2 tumors. Data are shown as mean ± SD. Statistical analysis was performed by one-way ANOVA. ***P < 0.001 and nsP > 0.05 indicate no statistical significance.

To further determine whether exosomal EPHA2-mediated angiogenesis is required for breast cancer metastasis in vivo, LM and HM breast cancer cells, control cells and EPHA2-silenced HM cells were subcutaneously injected into the fat pad of SCID mice to establish xenograft tumor models (Figure 6A). The inoculated tumors were similar in size (about 1 cm^3^) across all groups 30 days after inoculation. Then, luciferase-labeled T47D cells were injected into the xenograft tumor models through the tail vein. IVIS Spectrum imaging systems and computed tomography (CT) were performed periodically to observe the lung metastasis signal. After clear signals of lung metastases were observed by in vivo imaging, the mice were anesthetized, peripheral blood was collected, plasma was isolated, and exosomal EPHA2 protein content was measured by ELISA. The results showed that the level of exosomal EPHA2 was significantly elevated in the plasma from the HM and shControl groups compared with the LM and EPHA2-KD groups (Figure S3D). This result suggests that exosomal EPHA2 can be secreted into the peripheral blood circulation by HM breast cancer cells. Next, mice were sacrificed, and tumors were isolated. Measurements revealed comparable tumor sizes in each group (Figure S3E). IVIS Spectrum imaging showed that the fluorescence intensity of lung metastases in the HM group was significantly higher than that in the LM group, whereas the fluorescence intensity in the EPHA2 knockdown group was lower than that in the control group (Figure 6B). Consistently, similar results were observed in CT images (Figure 6C-Left). Moreover, the number of metastatic foci on the lung surface was significantly reduced in the EPHA2 knockdown group compared with the control group (Figure 6C-Middle). HE staining showed that the number of micro-metastases in lung tissue of mice inoculated with HM breast cancer cells was significantly higher than that in the group inoculated with LM breast cancer cells (Figure 6C-Right). Interestingly, the immunohistochemical microvessel density (MVD) measurement of primary tumors using anti-CD31 antibodies showed that the MVD in HM and control tumors was significantly increased compared with that in LM and EPHA2-silenced tumors (Figure 6D). Together, these results suggest that exosomal EPHA2 can promote the angiogenesis and metastasis of breast cancer cells in vivo.

### EPHA2 is an indicator of poor prognosis and progression in breast cancer

**Figure 7:**
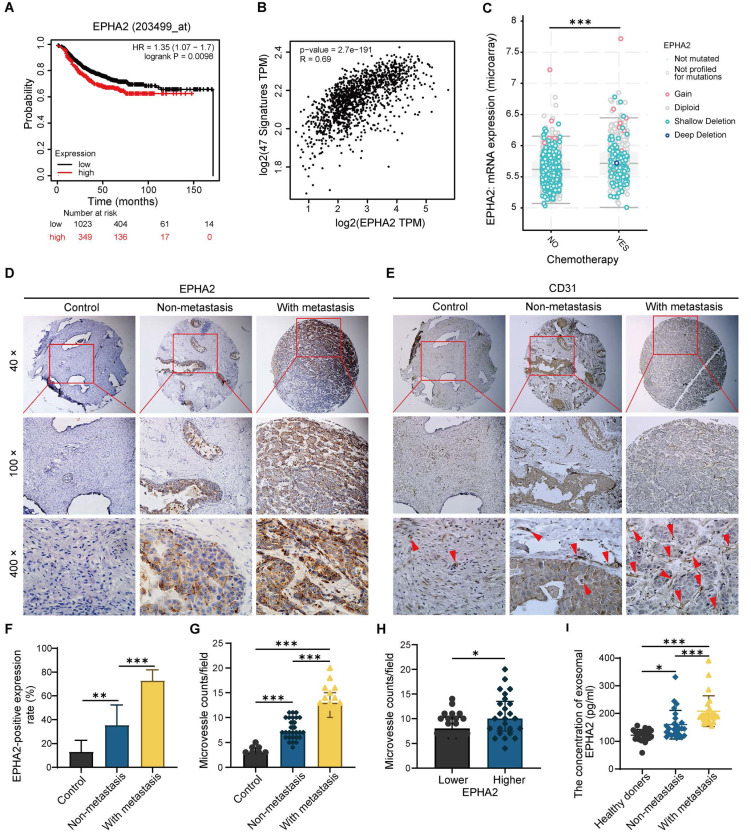
** EPHA2 is an indicator of poor prognosis and progression in breast cancer. A** Patients with high EPHA2 expression showed a lower survival rate in BRCA in the TCGA database. **B** TCGA data analysis suggested that EPHA2 expression was positively correlated with angiogenesis-related genes in BRCA. **C** The expression level of EPHA2 was higher in drug-resistant tissues in BRCA in the TCGA database. **D** Immunohistochemical staining to examine the expression of EPHA2 in a breast cancer tissue microarray. **E** Immunohistochemical staining to examine the expression of CD31 in a breast cancer tissue microarray. **F** The percentage score of EPHA2 in the paracancer group (Control), nonmetastatic group and metastatic group. **G** The number of CD31-stained microvesicles in the paracancer group (Control), nonmetastatic group and metastatic group. **H** The number of CD31-stained microvesicles in EPHA2 low expression and high expression tissues. **I** ELISA assays showed the concentration of exosomal EPHA2 in plasma collected from healthy donors (n=25), breast cancer patients without metastasis (n=25), or breast cancer patients with metastasis (n=25). Data are shown as mean ± SD. Statistical analysis was performed by one-way ANOVA. *P < 0.05, **P < 0.01, ***P < 0.001, and ns P > 0.05 indicate no statistical significance.

To further determine the relationship between EPHA2 expression and the prognosis of BRCA, we analyzed the expression levels of EPHA2 in BRCA using TCGA database. We found that patients with high EPHA2 expression showed a lower survival rate (Figure 7A). In addition, EPHA2 expression was positively correlated with the expression of angiogenesis-related genes (Figure 7B), and the expression levels of EPHA2 were higher in drug-resistant tissues (Figure 7C). These data suggest that elevated EPHA2 expression is associated with breast cancer progression. To further determine the role of EPHA2 in breast cancer patients, immunohistochemical staining was used to examine the expression of EPHA2 and CD31 in a breast cancer tissue microarray, including 8 cases of paraneoplastic tissues, 28 cases of non-metastatic tissues and 19 cases of metastatic breast cancer tissues. The data showed that the expression of EPHA2 was higher in breast cancer tissues than in paracancer tissues. Notably, the expression of EPHA2 was significantly higher in metastatic breast cancer compared with non-metastatic breast cancer (Figure 7D-F). We further examined the MVD in tissues by using CD31 labeling, a biomarker of vascular cells. As we expected, the number of microvessels in the breast cancer tissues was significantly higher than that in the paracancer tissues. Moreover, the number of microvessels in metastatic breast cancer tissues was significantly higher than that in non-metastatic breast cancer tissues (Figure 7E-G). In addition, the breast cancer tissues were divided into the low- and high-expression groups according to the expression levels of EPHA2. We found that CD31 intensity was significantly higher in tissues with high EPHA2 expression, indicating a strong positive correlation between EPHA2 protein expression and angiogenesis (Figure 7H). We further collected plasma from healthy donors and breast cancer patients (with and without metastasis). The exosomes were extracted, and the levels of exosomal EPHA2 were determined by ELISA. The results showed that the levels of exosomal EPHA2 were significantly higher in plasma from breast cancer patients than those from healthy blood donors. Moreover, the plasma levels of exosomal EPHA2 were significantly higher in patients with metastatic breast cancer than in those with non-metastatic breast cancer (Figure 7I). Hence, these results suggest that a high level of EPHA2 in circulating exosomes is an indicator of breast cancer metastasis.

## Discussion

**Figure 8:**
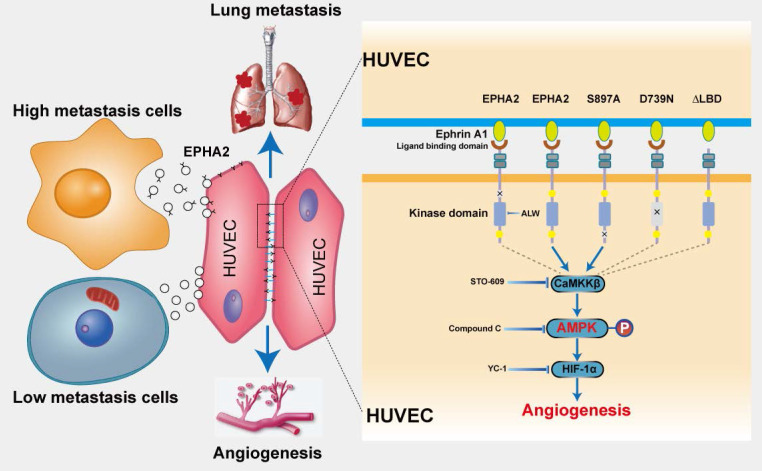
** Schematic model of the proposed mechanisms of this study.** Compared with LM breast cancer cells, the exosomes released by HM breast cancer cells are rich in EPHA2. Exosomal EPHA2 is delivered from HM breast cancer cells to endothelial cells and mediates activation of the Ephrin A1-EPHA2 forward signaling, which further activates the downstream AMPK-HIF-1α signaling pathway and promotes angiogenesis.

Tumor metastasis is a complex multistep process, which requires not only needs tumor cell reprogramming to achieve high metastasis but also remodeling the microenvironment of the distal site to establish a metastatic niche suitable for tumor cell colonization and growth 28-30. Angiogenesis plays a key role in the establishment of metastatic niches 31-33. To promote angiogenesis, tumor cells communicate with stromal cells and educate them to “follow their footsteps,” facilitating the invasion of tumor cells into local circulation to colonize distal regions 34-36. Exosomes secreted by tumor cells mediate cell-cell communication in the cancer microenvironment 37-40. Herein, we identified a novel model, in which EPHA2-rich exosomes derived from HM breast cancer cells can promote the angiogenic potency of endothelial cells. Exosomal EPHA2 can be transferred from HM breast cancer cells to endothelial cells to mediate the upregulation of EPHA2 in endothelial cells. EPHA2 activates AMPK signaling via the ligand Ephrin A1-dependent forward pathway to promote the tube-forming and migration ability of endothelial cells, thereby promoting breast cancer metastasis (Figure 8). Our work highlights the critical functional role of exosomal EPHA2 in promoting angiogenesis via mediating the communication between tumorigenic cancer cells and microenvironmental endothelial cells. Our findings also suggest that the increased exosomal EPHA2 in the circulation may be an important mechanism for accelerating breast cancer progression and an indicator of tumor metastasis.

The exosome-mediated interaction between cancer cells and stromal cells (such as macrophages, fibroblasts and Treg cells) in the tumor microenvironment promotes angiogenesis and accelerates tumor progression 41, 42. Exosomes secreted by cancer cells also promote angiogenesis 43-45. However, the function and mechanism of heterogeneous cancer cells in tumors in regulating angiogenesis remain unclear. In this study, we found that HM-Exos can not only promote the migration and tube formation ability of endothelial cells in vitro, but also enhance the angiogenesis of primary tumors in vivo. These data suggest that HM breast cancer cells are not only the seeds of tumor metastasis, but can also facilitate metastasis by domesticating endothelial cells, promoting angiogenesis and establishing a favorable metastatic niche. Moreover, although LM-Exos can promote lung metastasis than the control group, the micrometastatic burden in the lung tissue of mice induced by HM-Exos was more obvious than that of mice in the LM-Exos group. Interestingly, recent studies have shown that HM hepatocellular carcinoma (HCC) cells can enhance the invasion and metastasis of low metastatic HCC cells through exosomes 17. In addition, exosomes transfer from highly metastatic cells to poorly metastatic cells, accelerating the progression of nasopharyngeal carcinoma 16. Hence, HM cancer cells endow LM cells with invasive ability, and their ability to promote angiogenesis makes them more effective in metastasis. Collectively, these results indicated that highly metastatic subpopulations amongst heterogeneous tumor cells are the key factor in determining tumor progression.

One of our key findings is that EPHA2 enriched in HM-Exos mediates the pro-angiogenetic effect. As a receptor tyrosine kinase located on the cell membrane, EPHA2 interacts with the cell surface anchoring ligand Ephrin A1 to form a critical cell-cell communication system. Thus, Ephrin A1-EPHA2-mediated signal transduction requires direct cell-cell contact. Recent studies have shown that Ephs proteins can be secreted into exosomes 46. However, the expression and function of EPHA2 on tumor cell-derived exosomes have received little attention. Herein, we found that EPHA2 is highly expressed in HM breast cancer cells and exosomes. Moreover, EPHA2-rich exosomes significantly promoted the migration and tube formation of endothelial cells in vivo and enhanced the outgrowth of rat arterial ring ex vivo, whereas exosomes containing lower EPHA2 or Exos from EPHA2 stable silenced cells lost these effects. These data suggest that EPHA2 expressed on exosomes is necessary for angiogenesis. Unlike our previous report 20, we found that exosomal EPHA2 can be internalized and expressed by endothelial cells, which express high levels of Ephrin A1. Interestingly, EPHA2-rich exosomes could still promote angiogenesis in EPHA2-silenced endothelial cells. Hence, exogenous EPHA2 carried by exosomes is the main contributor to endothelial cell angiogenesis. Collectively, our findings indicate that exosomal EPHA2 released by HM breast cancer cells may educate endothelial cells through paracrine action, promote angiogenesis and facilitate the establishment of a long-distance niche suitable for metastasis.

To date, little information is available on the detailed mechanism by which exosomal EPHA2 enhances angiogenesis. In view of the fact that exosomal EPHA2 can be endocytosed and expressed by endothelial cells, EPHA2 can theoretically function in a kinase-dependent or independent manner. Herein, exosomes carrying wild-type EPHA2 or EPHA2-S897A mutant, but not the kinase-deficient EPHA2-D739N mutant, promoted the tube formation of endothelial cells and the outgrowth of rat arterial ring. In addition, endothelial cells treated with EPHA2-rich exosomes showed an apparent increase in EPHA2 Tyr588 phosphorylation, which is an indicator of EPHA2 kinase activation. Moreover, blocking EPHA2 kinase activity by using inhibitors significantly inhibited the pro-angiogenic effect of exosomal EPHA2. These data suggest that exosomal EPHA2 promotes angiogenesis depending on its kinase activity. The kinase activity of EPHA2 is always related to the Ephrin A1-dependent forward signaling pathway 47. Consistent with this hypothesis, exosomes carrying EPHA2-ΔL lost the pro-angiogenic effect. Moreover, EPHA2-rich exosomes failed to promote the angiogenesis of Ephrin A1-silenced endothelial cells. These exosomes also failed to increase EPHA2 Tyr588 phosphorylation in Ephrin A1-KD cells. Hence, exosomal EPHA2 promotes angiogenesis through kinase- and ligand binding-dependent forward signaling pathways. Consistently, a previous study has shown that Ephrin A1 activating EPHA2 on endothelial cells promotes angiogenesis 48. We previously showed that exosomal EPHA2 confers the invasive phenotype transfer from drug-resistant cells to sensitive cells through the ligand Ephrin A1-dependent reverse pathway 20. These findings demonstrated that exosomal EPHA2 can affect tumor cells and stromal cells in different ways and accelerate tumor progression.

Although we have demonstrated that the activation of forward signaling is required for the pro-angiogenic effect of EPHA2, this finding is distinct from the results of previous studies on tumor cells, in which Ephrin A1-dependent activation of EPHA2 inhibits the proliferation and migration of cancer cells 49, 50. This may be due to different downstream signaling pathways activated by EPHA2 in distinct cell types. Therefore, the detailed molecular mechanisms downstream of Ephrin A1-EPHA2 forward signaling in endothelial cells need to be further investigated. Herein, GSEA of RNA-Seq data revealed that the activation of AMPK pathway was significantly induced in EPHA2-rich-exosome-treated cells. Consistently, the level of p-AMPK was significantly higher in the EPHA2-rich-exosome-treated endothelial cells, whereas the phosphorylation levels of other key signal molecules were not altered. Moreover, exosomes from EPHA2-silenced cells failed to induce an increase in p-AMPK. These data suggest an involvement of AMPK downstream of Ephrin A1-EPHA2 forward signaling. In addition, in Ephrin A1-silenced endothelial cells, exosomal EPHA2 could not induce an elevation in phosphorylation of AMPK, further supporting the role of AMPK downstream of Ephrin A1-EPHA2 signaling. AMPK is known as a cellular energy regulator 51, 52. Several studies have shown that AMPK is involved in regulating the function of vascular endothelial cells and angiogenesis under pathological conditions. AMPK activation can upregulate the expression of pro-angiogenic factors such as HIF-1α to promote angiogenesis and improve local blood supply and oxygenation in cardiovascular and cerebrovascular disease models 27, 53. Consistently, the inhibition of AMPK by compound C or STO609 blocked the upregulation of AMPK phosphorylation induced by EPHA2-rich exosomes, as well as tube formation of endothelial cells in vitro and vascular growth of rat arterial rings. In addition, compound C also inhibited the upregulation of HIF-1α by EPHA2-rich exosomes. Likewise, HIF1-α silencing or its inhibition abolished the pro-angiogenic effect of EPHA2-rich exosomes on endothelial cells. Collectively, our results suggest that exosomal EPHA2 derived from HM breast cancer cells promotes breast cancer progression through the AMPK-HIF-1α pathway downstream of Ephrin A1-EPHA2 forward signaling.

In conclusion, our findings demonstrated a critical role of EPHA2-rich exosomes in promoting angiogenesis and metastasis of breast cancer. Exosomal EPHA2 transferred from HM breast cancer cells to endothelial cells confers the pro-angiogenic effect by activating AMPK signaling through a ligand Ephrin A1-dependent forward pathway. Thus, our study identifies a novel mechanism of exosomal EPHA2-mediated intercellular communication from breast cancer cells to endothelial cells in the tumor microenvironment to provoke angiogenesis and metastasis. High levels of EPHA2 in circulating exosomes are associated with cancer progression and suggest poor clinical outcomes in breast cancer patients. Thus, EPHA2 is a potentially powerful biomarker for poor prognosis and progression of breast cancer, and targeting exosomal EPHA2-AMPK signaling may serve as a potential strategy for breast cancer therapy.

## Materials and Methods

### Collection of human blood samples

With the patients' informed consent, all plasma samples were collected from Tianjin Medical University Cancer Institute and Hospital. Finally, blood samples (plasma) of 25 breast cancer patients with metastasis, 25 breast cancer patients without metastasis and 25 plasma samples from healthy age- and gender-matched volunteers were collected. Plasma samples were centrifuged at 1000 g for 10 min and stored at -80°C. Table 1 summarizes the clinicopathological characteristics of the patients who participated in this study. This study followed the ethical guidelines of the “Declaration of Helsinki” in 1975, and the protocol was approved by the Ethics Committee of Tianjin Medical University Cancer Institute and Hospital.

### Cell culture

Human umbilical vein endothelial cells (HUVECs, Shanghai Gefan Biotechnology Co., Ltd.) were cultured in F12K medium (Gibco, USA). T47D, MDA-MB-231 and BT549 cells were cultured in RPMI-1640 medium (HyClone, Logan, UT, USA). MDA-MB-468 cells were cultured in DMEM/F12 medium (HyClone, Logan, UT, USA). HEK-293T cells were cultured in DMEM high-glucose medium (HyClone, Logan, UT, USA). All media were supplemented with 10% fetal bovine serum (FBS, Gibco, Carlsbad, CA, USA) and 1% penicillin and streptomycin. All cells were cultured in a humidified incubator at 37°C and 5% CO_2_.

### Isolation of exosomes from medium and serum

To minimize the influence of FBS exosomes on cell functions, we first removed the FBS exosomes by ultracentrifugation at 100,000 g for 16 h and then aseptically filtered them with a 0.22 μm filter. The exosomes were isolated from the exosome-free cell culture medium by sequential differential centrifugation after 48 h of culture, as previously described 54. In short, the cell culture medium was first centrifuged at 300 g and 3000 g for 10 min to remove cells and other debris, and the supernatant was centrifuged at 10,000 g for 30 min to remove large vesicles. Finally, the supernatant was centrifuged at 100,000 g for 90 min (all steps were performed at 4°C). The pure exosomes were concentrated as pellets and resuspended in PBS. The exosomes in human plasma samples were isolated as described by Kahlert et al 55, 56. In brief, 500 μL of plasma samples were thawed on ice. The plasma was diluted with PBS and then ultracentrifuged at 160,000 g overnight. Next, the exosomal precipitate was washed in PBS and then subjected to second step ultracentrifugation at 160,000 g for 2 h (all steps were performed at 4°C). The pure exosomes were concentrated as pellets and resuspended in 100 μL of PBS.

### Exosome preparation and characterization

Transmission electron microscopy (TEM) and multiparametric nanoparticle optical analysis (NanoSight) were used to determine the size and shape of the exosomes. Exosome-specific proteins were identified by Western blot with anti-CD81 antibody (56039s, CST, MA, USA), anti-Alix antibody (92880, CST, MA, USA) and TSG101 antibody (sc-136111, Santa Cruz, CA, USA). Western blot was performed with calnexin antibody (sc-23954, Santa Cruz, CA, USA) as negative control to monitor cytoplasm contamination.

### Exosomal transfer detection

Exosomes were stained with the PKH26 Red Fluorescent Cell Linker Kit (Sigma-Aldrich, MO, USA) according to the manufacturer's instructions. Firstly, exosomes were suspended in 250 μL of diluent C and then well mixed with 250 μL of diluent C containing 1 μL of PKH26 dye. After incubation at room temperature for 5 min, 200 μL of the serum was added and incubated for 1 min to stop staining. Finally, the stained exosomes were diluted in PBS and ultracentrifuged at 100,000 g at 4°C for 90 min, then resuspended in F12K medium containing 10% FBS and co-cultured with HUVECs in a humidified incubator at 37°C for 24 h. After incubation, the cells were washed three times with PBS. Then, the cells were fixed with 4% paraformaldehyde for 30 min, washed three times with PBS and counterstained by DAPI for 10 min. The cells were observed under a confocal laser scanning microscope (IX82, Olympus, Japan).

### Enzyme-linked immunosorbent assay (ELISA)

Plasma exosomal EPHA2 levels were assessed using the Human EPHA2 ELISA Kit (Jianglai Biological, China, Cat no.: JL19621) according to the manufacturer's instructions.

### Western blot

Western blot was performed as described previously 57. Briefly, whole-cell lysates or exosomal proteins were separated and transferred onto polyvinylidene fluoride membranes by sodium dodecyl sulfate-polyacrylamide gel electrophoresis. After sealing with 5% skim milk in TBST, the membrane was incubated with corresponding primary antibodies. The following antibodies were used: AMPK (2532, CST), p-AMPK (2535, CST), mTor (2972, CST), p-mTor (2971, CST), ERK (4695, CST), p-ERK (4370, CST), Akt (9272, CST), p-Akt (2965, CST), EPHA2 (6997, CST), p-EPHA2(Tyr588) (12677,CST), Ephrin A1 (124911, Abcam) and β-actin (A1978, Sigma-Aldrich, MO, USA). After washing three times with TBST, the membranes were incubated with HRP-conjugated secondary antibody (Bio-Rad, USA, Cat no.: 1706515 or 1706516 ) for 1 h at room temperature. Protein bands were detected using an ECL kit (Millipore) according to the manufacturer's instructions.

### HUVEC tube formation assay

For the tube formation assay, 50 μL of Matrigel (BD Growth Factor Reduced Matrigel Matrix) was added to each well of a 96-well plate and allowed to polymerize at 37°C for 30 min. Next, cells were resuspended in F12K medium without FBS and inoculated in each well at a concentration of 2 × 10^4^ cells/well. After 4 h, the cell structure on the matrix gel was analyzed by an inverted optical microscope (Nikon). All tubular structures of each well were counted. Only closed tubular networks were calculated. These closed tubular networks represent the degree of angiogenesis in vitro.

### Rat arterial ring budding experiment

Six-week-old Sprague-Dawley rats were sacrificed by anesthesia, and the aorta was dissected and washed. The periaortic fat was separated by immersion in sterile PBS, and the aorta was cut into 1-1.5-mm-thick sections and placed in a 96-well plate. About 70 μL of Matrigel was added to each well and incubated at 37°C for 1 h to solidify the gel. Then, it was added with 150 μL of DMEM high-glucose medium with or without exosomes (including 10% FBS, 100 U/mL penicillin and 100 mg/mL streptomycin). The condition of the vascular rings was observed daily. When a large number of cell divergent growths such as microvessel structures appeared around the vascular ring, photographs were taken under a phase contrast microscope, and the number of microvessels was counted and then statistically analyzed.

### Matrigel plug assay and CD31 immunohistochemical staining

Eight- to 10-week-old C57BL/6 female mice were injected subcutaneously in the groin with 0.6 mL of Matrigel mix (0.1 mL of PBS suspension containing exosomes (200 μg/mL) + 0.5 mL of Matrigel (354234 Corning) + 50 units of heparin). The negative control group was 0.1 mL of PBS buffer + 0.5 mL of Matrigel + 50 units of heparin. The positive control group was 0.1 mL of PBS buffer + 0.5 mL of Matrigel + 50 units of heparin + 100 ng/mL of VEGF (450-32 Peprotech). After 1 week, the mice were executed, the skin was incised, the Matrigel plugs were peeled off, the morphology of Matrigel and the angiogenesis within the gel were observed, the Matrigel plugs were cut and fixed in 4% formaldehyde, and the sections were routinely embedded in paraffin for subsequent experiments. Sections were stained with anti-CD31 antibody (BD Biosciences). CD31 immunohistochemical staining was used to detect CD31+ endothelial cells in the Matrigel plug. Most angiogenesis occurs at the edge of the arterial plug, growing inward toward the center of the plug. Micrographs of CD31-stained sections were produced, and morphometric image analysis was performed. ImageJ software was used to quantify angiogenesis.

### Wound healing and Transwell assay

Wound healing assay and Transwell (Corning, NY, USA) migration assay were used to determine the migration capacity of HUVECs. In the wound healing assay, cells were inoculated in 6-well plates. After 24 h, each well was scraped with a 10 μL pipette tip to create a linear region without cells and added with medium containing exosomes in 1% FBS. The culture plates were then incubated at 37°C in 5% CO_2_ for 24 h. The width of the wound gap was observed and photographed under an inverted microscope. For the Transwell assay, pretreated HUVECs were transferred to the upper chamber in 200 μL of serum-free growth medium (5 × 10^4^ cells/8.0 μm pore size of Boyden chambers). In addition, 500 μL of medium containing 10% FBS was added to the lower chamber. For cell invasion assay, 5 × 10^4^ cells suspended in 200 μL of serum-free medium were loaded into the upper chamber coated with Matrigel. After being cultured at 37°C for 12 h, the migrating or invading cells were fixed and stained, and the number of invading cells was counted under an optical microscope.

### Immunofluorescence analysis

Cells were inoculated in 12-well plates containing glass coverslips and incubated in 5% CO_2_ at 37°C for 24 h. Subsequently, after fixation in 4% paraformaldehyde and permeabilization in 0.1% Triton X-100, the cells were sealed in 3% BSA solution. Cells were then incubated with primary antibody at 4°C overnight. After washing three times with PBS, the cells were stained with Alexa Fluor 488-conjugated secondary antibody (ThermoFisher, USA, Cat no.: 710369) for 1 h at room temperature and protected from light, followed by nuclear staining with 1 ng/mL of DAPI. Coverslips were observed by using a laser scanning confocal microscope (Zeiss Axio Imager).

### Vector construction and stable transfection

EPHA2 and Ephrin A1 specific shRNA sequences were subcloned into the lentiviral vector pLKO.1-puromycin (Addgene, USA, Cat no.: 8453) at the BamHӀ and AgeӀ cloning sites. The sequences of the shRNAs are listed in Table S1. Polymerase chain reaction (PCR) was used to clone the EPHA2 coding sequence from a cDNA plasmid purchased from ORIGENE (Beijing, China). Truncated mutants EPHA2-ΔL with mCherry markers were created by overlapping PCR and cloned into linearised pCDH vectors (Addgene, USA, Cat no.: 72265) using the ClonExpress II one-step cloning kit (Vazyme Biotech, Nanjing, China). The mCherry-tagged EPHA2 (D739N) and EPHA2 (S897A) point mutations were introduced by PCR-based site-directed mutagenesis and cloned into the pCDH vector. All plasmids were confirmed by restriction enzyme digestion and DNA sequencing. The primers used for EPHA2 and its mutants are listed in Table S2.

### Cellular RNA sequencing analysis and bioinformatics analysis

For transcriptome sequencing (RNA-Seq), RNA was prepared from three biological replicates of HUVECs treated with EPHA2-rich exosomes and control exosomes. The pooled RNA for each experimental condition was made by mixing the Trizol extracts from three biological replicates. The pooled RNA was divided into 3 aliquots and subjected to libraries generation and sequencing. Then, the RNA samples were processed and sequenced by NovoGene (Beijing, China), the details for RNA-Seq is described in Supplementay Method. Briefly, the Illumina TruSeq mRNA Prep Kit and NEB Next Ultra RNA Library Prep Kit for Illumina were used to construct the libraries and the reads were filtered by Q30 (Table S3-4 and Figure S5). Gene set enrichment analysis (GSEA) was performed using the WebGestalt database (http://www.webgestalt.org/) as previously described 58. Gene Ontology (GO) and Kyoto Encyclopedia of Genes and Genomes (KEGG) enrichment analysis were performed using clusterProfiler R package, the differently expressed genes were as input and gene length bias was corrected, P values less than 0.05 were significantly enriched by differential expressed genes (Table S5). In addition, the correlation between EPHA2 and 47 angiogenesis-related genes was analyzed using a subfunction of the GEPIA database (GEGIA, gene signature correlation in correlation analysis section). The 47 angiogenic-related genes were obtained in MisigDB under the HALLMARK geneset (Table S6).

### In vivo transfer assay

Four-week-old female SCID mice were purchased from Charles River (Beijing, China). All animal work procedures were approved by the Animal Ethical and Welfare Committee of Tianjin Medical University Cancer Institute and Hospital. Mice were randomly divided into five groups (6 mice/group). A total of 5 × 10^6^ cells (T47D, MDA-MB-231, control and EPHA2 stable knockout MDA-MB-231 cells) were inoculated subcutaneously into immunodeficient mice. After injection, the body weight and tumor size of mice were measured once a week, and the subcutaneous tumor volume was calculated by the standard modified formula volume (cm^3^) =1/2 (height ^2^ × length). A total of 1 × 10^6^ Luciferase-labelled T47D cells were injected into SCID mice via the tail vein, and IVIS Spectrum imaging system (PerkinElmer, USA) was used to observe the occurrence of lung metastasis of T47D cells in each group of tumor-bearing mouse models. One month after injection, the mice were anesthetized, and their peripheral blood was collected. Then, the mice were executed, and lung tissue was dissected and fixed in 4% neutral buffered formalin. Then, paraffin-embedded tissues were stained by HE and immunohistochemistry. Metastatic nodules were counted by HE staining.

### Tissue microarray analysis

Formalin-fixed paraffin-embedded human breast cancer tissue microarrays were obtained from Shanghai SuperChip Biotech (HBreD055CD01). Microarrays of 55 human tissues were divided into paraneoplastic (n = 8) and breast cancer groups (n = 47). The breast cancer groups were further divided into non-metastatic (n = 28) and metastatic groups (n = 19) based on the occurrence of metastasis. IHC detected the expression of CD31, EPHA2 and Ephrin A1 in tissue microarray. Tissue microarrays were incubated with EPHA2 (6997, CST), Ephrin A1 (124911, Abcam) and CD31 (BD Biosciences). To ensure unbiased results, data were collected in a double-blind manner. Antibody expression was scored by staining intensity. We divided the staining intensity into four groups: 0 (-), 1 (+), 2 (++) and 3 (+++). (-), (+), (++) and (+++) were defined as no staining, weak staining, moderate staining and strong staining. 0 (-) and 1 (+) were defined as low expression; 2 (++) and 3 (+++) were defined as high expression.

### Statistical analysis

All data are expressed as mean ± SD of at least three independent experiments. Statistical analysis was performed using GraphPad Prism 8.0 software (GraphPad Software, USA). One-way or two-way ANOVA was performed for differences between groups. P < 0.05 was considered statistically significant.

## Supplementary Material

Supplementary methods, figures and tables 1-4.

Supplementary table 5.

Supplementary table 6.

## Figures and Tables

**Table 1:** Clinicopathological characteristics of breast cancer patients included in this study

Characteristic	Healthy group (n=25)	Breast cancer group (50)	
		Non-metastasis (n=25)	With metastasis (n=25)
**Median age at diagnosis (years)**	46 (25-73)	50 (31-72)	52 (34-69)
**Gender**			
Male	0	0	0
Female	25	25	25
**Histologic grade**			
I-II	0	25	8
III-IV	0	0	17
**Tumor stage**			
T1	0	20	6
T2-T4	0	5	19
**Lymph Node stage**			
N0-N1	0	24	7
N2-N3	0	1	18
**Metastasis**			
M0	0	25	0
M1	0	0	25
**Chemotherapy**			
Received	0	3	25
Not received	0	22	0

## Abbreviations

HM: High metastatic potential
LM: Low metastatic potential (LM)
Exos: Exosomes (Exos)
ELISA: Enzyme linked immunosorbent assay (ELISA)
NTA: Nanoparticle tracking analysis (NTA)
KEGG: Kyoto Encyclopedia of Genes and Genomes
GO: Gene Ontology
GSEA: Gene set enrichment analysis
FDR: False Discovery Rate
