# Supplementary material for: Exosomal EPHA2 derived from highly metastatic breast cancer cells promotes angiogenesis by activating the AMPK signaling pathway through Ephrin A1-EPHA2 forward signaling
Source: Theranostics. 2022 May 13;12(9):4127–46. doi: 10.7150/thno.72404 (PMC9169374; doi:10.7150/thno.72404)
Supplement: Supplementary file 1 — Supplementary methods, figures and tables 1-4. [file thnov12p4127s1.pdf]

# Supplementary Materials

## Supplementary Methods

### HIF-1α knockdown or Inhibition

HIF-1α siRNAs were obtained from GenePharma (163971 and 163972, Suzhou China). The HIF-1α antibody was obtained from Abcam (51608, Cambridge, USA). The HIF-1α inhibitor 3-(5′-hydroxymethyl-2′-furyl)-1-benzylindazole (YC-1) was obtained from MedChem Express (Princeton, NJ, USA). Transfections were performed using Lipofectamine™ RNAiMAX Transfection Reagent (ThermoFisher, 13778150) according to the manufacturer's instructions. After 48 h of transfection, cells were then treated with the indicated exosomes and HUVECs were further analyzed for their tube-forming ability.

### RNA sequencing and Bioinformatics

**Sample collection and preparation**

**RNA quantification and qualification**

RNA integrity was analyzed using the RNA Nano 6000 Assay Kit of the Bioanalyzer 2100 system (Agilent Technologies, CA, USA).

**Library preparation**

Total RNA was extracted using Trizol (ThermoFisher, USA, Cat no.: 710369) as input for the RNA isolation. Briefly, mRNA was purified from total RNA using poly-T oligo-conjugated magnetic beads. The enriched mRNAs were fragmented using divalent cations under elevated temperature in the First Strand Synthesis Reaction Buffer(5X). First strand cDNA was synthesized using random hexamer primers and

M-MuLV Reverse Transcriptase, followed by RNA degradation using RNaseH Second strand cDNA synthesis was subsequently performed using DNA Polymerase I and dNTPs. The remaining overhangs were converted into blunt ends via exonuclease/polymerase activities. After adenylation of 3' ends of DNA fragments, Adapters with hairpin loop structures were ligated to prepare for hybridization. In order to select cDNA fragments of preferentially 370~420 bp in length, the library fragments were purified using the AMPure XP system (Beckman Coulter, Beverly, USA). Then PCR was performed with Phusion High-Fidelity DNA polymerase, Universal PCR primers and Index (X) Primer. At last, PCR products were purified (AMPure XP system) and library quality was assessed on the Agilent Bioanalyzer 2100 system.

**Clustering and sequencing**

The clustering of the index-coded samples was performed on a cBot Cluster Generation System using TruSeq PE Cluster Kit v3-cBot-HS (Illumia) according to the manufacturer's instructions. Then library preparations were sequenced on an Illumina Novaseq platform and 150 bp paired-end reads were generated.

**Data Analysis**

**Quality control**

Raw data (raw reads) of fastq format were firstly processed to obtain clean data (clean reads) by removing reads containing adapter, reads containing N base and low quality reads. In this step, the Q20, Q30 and GC content the clean data were calculated. The clean data were used in downstream data analysis.

**Reads mapping and Quantification**

Reference genome (hg38) and gene model annotation files were downloaded directly from the genome website . Indexing and paired-end clean reads alignment was preformed using Hisat2 v2.0.5. FeatureCounts v1.5.0-p3 was used to count the reads numbers mapped to each transcripts, and the FPKM of each gene was calculated.

**Differential expression analysis**

Differential expression analysis of two conditions/groups (three biological replicates per condition) was performed using the DESeq2 R package (1.20.0). The P-values were adjusted using the Benjamini and Hochberg's approach for controlling the false discovery rate. Genes with adjusted P-values $<0.05$ found by DESeq2 were assigned as differentially expressed.

**Enrichment analysis of differentially expressed genes**

Gene Ontology (GO) enrichment analysis of differentially expressed genes was implemented by the clusterProfiler R package, in which gene length bias was corrected. GO terms with corrected P-value less than 0.05 were considered significantly enriched by differential expressed genes.

# Supplementary Figures

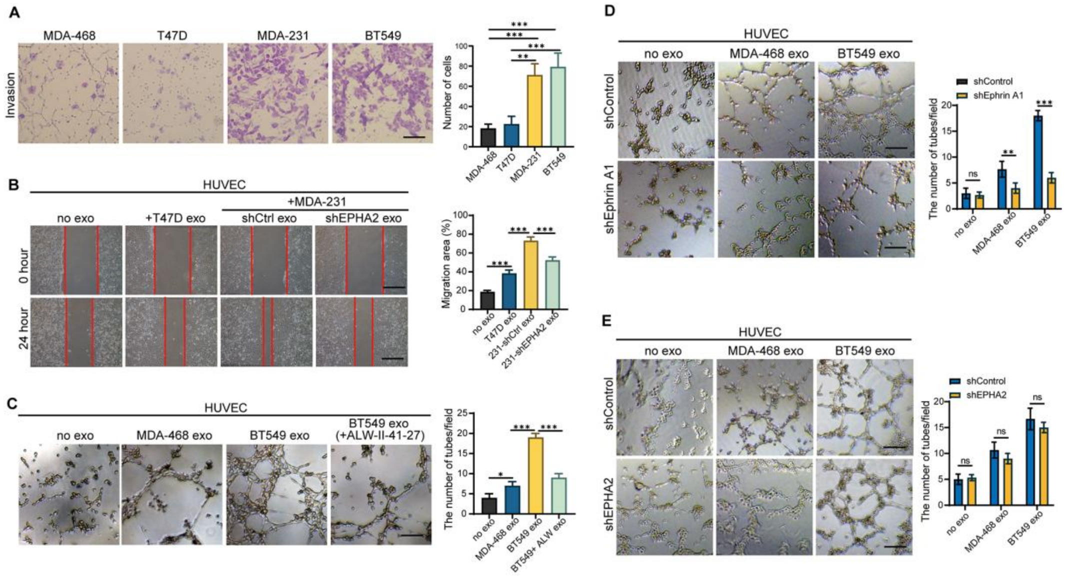


**Figure S1**

**A** The invasion ability of breast cancer cells with high metastatic potential (MDA-231 or BT-549) was significantly higher than that of breast cancer cells with low metastatic potential (MDA-468 or T47D). For cell invasion assay, $1 \times 10^5$ cells suspended in 200 μL of serum-free medium were loaded onto the upper chambers coated with Matrigel. The incubation time was 24 h. The statistical results were summarized in the right panel. **B** EPHA2-silenced HM-exos failed to promote the migration of endothelial cells. **C** The inhibitor-treated HM-Exos could not promote the tube formation of endothelial cells. **D** HM-Exos failed to promote the tube-forming ability of Ephrin A1-KD endothelial cells. **E** HM-Exos still promote the tube-forming ability of EPHA2-KD endothelial cells. Data were expressed as mean ± SD. All experiments were repeated at least three times. $^*P < 0.05$, $^{**}P < 0.01$, $^{***}P < 0.001$ and ns $P > 0.05$ indicate no statistical significance. Scale bar: 200 μm.

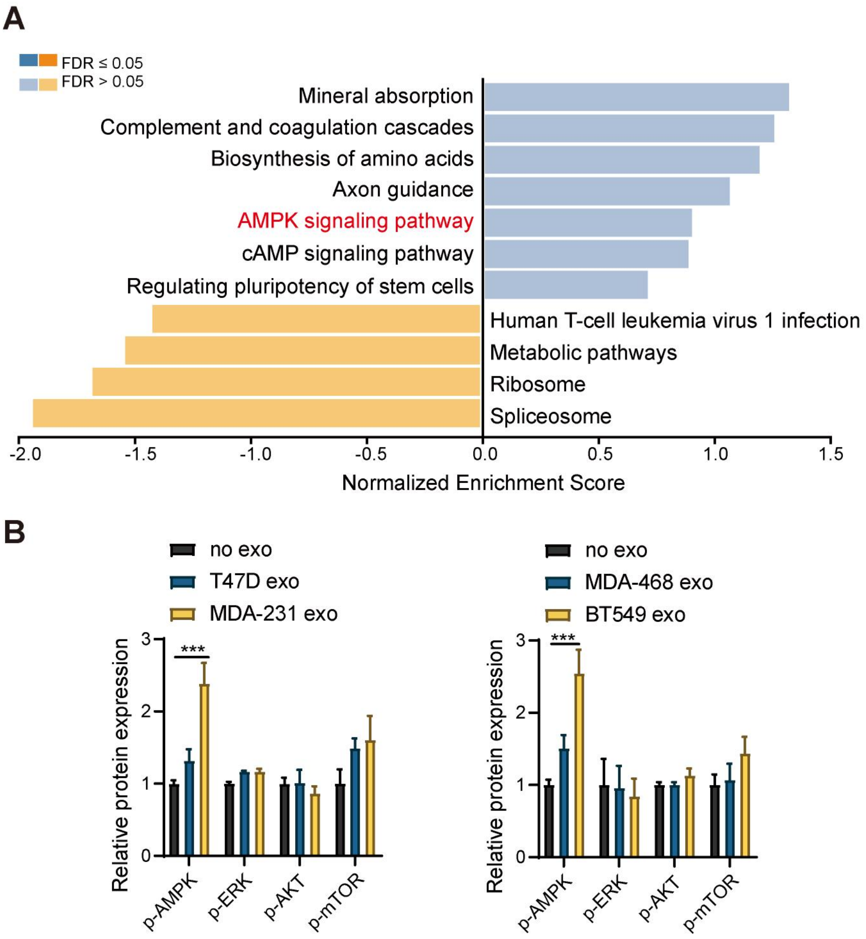


**Figure S2**

**A** Gene set enrichment analysis (GSEA) of the RNA-Seq data showed that the AMPK signaling pathway was induced in EPHA2-rich exosomes treated HUVECs compared with control exosome treated cells. FDR, False Discovery Rate. **B** Quantification of the western bloting images in Figure 5B. The analysis was performed using WB scans from three biological repeats.

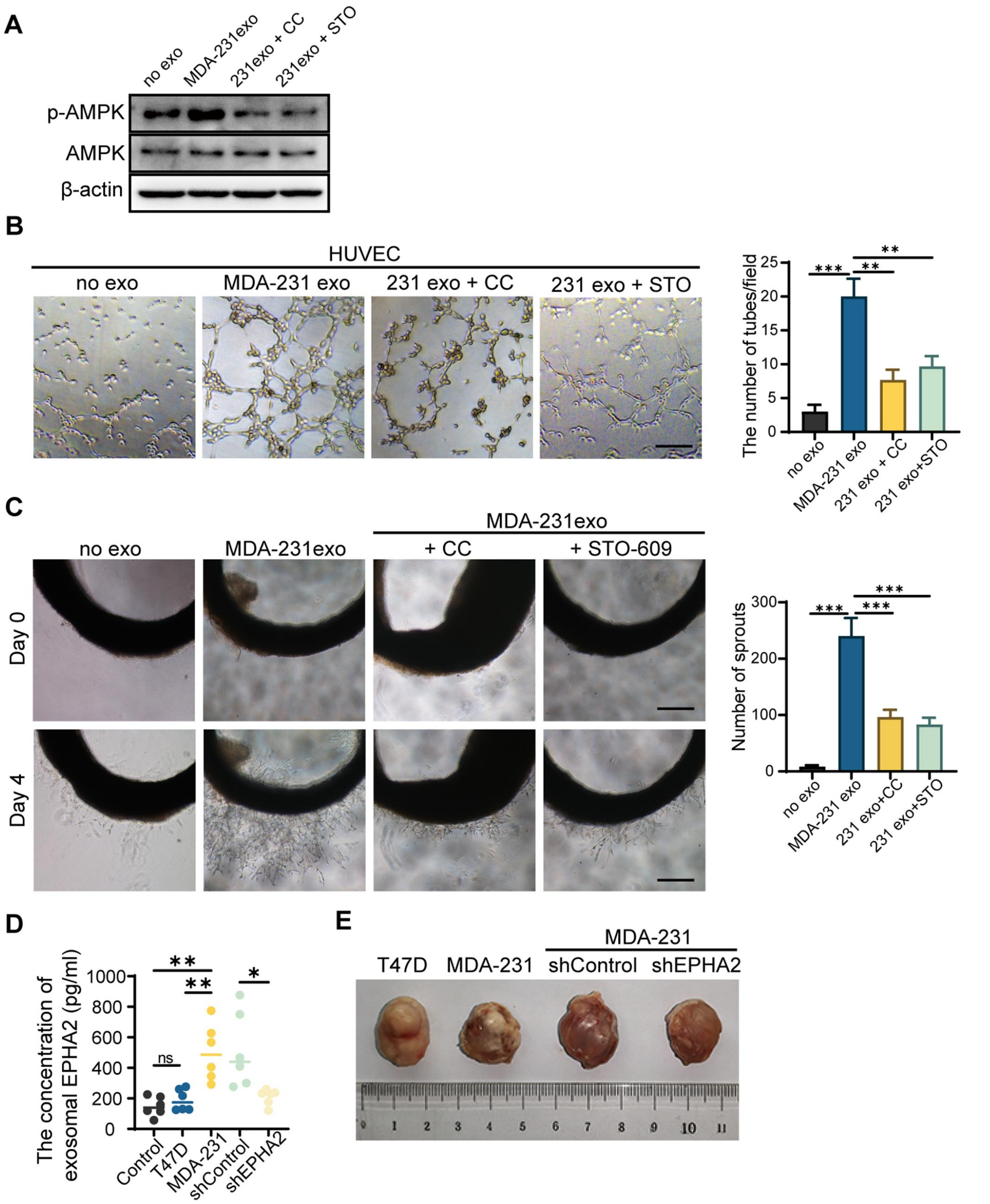


**Figure S3**

**A** Compound C or STO609 eliminated the phosphorylation of AMPK in HUVEC that incubated with HM-Exos. **B, C** Inhibition of AMPK signaling by Compound C or STO609 reduced the tube-forming and rat arterial ring outgrowth capacity in HM-Exos treated endothelial cells. **D** Exosomal EPHA2 was significantly upregulated in plasma of mice from the MDA-231 and MDA-231-shControl groups compared

with the T47D and MDA-231-shEPHA2 groups. **E** Representative images of subcutaneous tumor formed in mice. All experiments were repeated at least three times. *$P < 0.05$, **$P < 0.01$, ***$P < 0.001$ and ns $P > 0.05$ indicate no statistical significance. Scale bar: 200 μm.

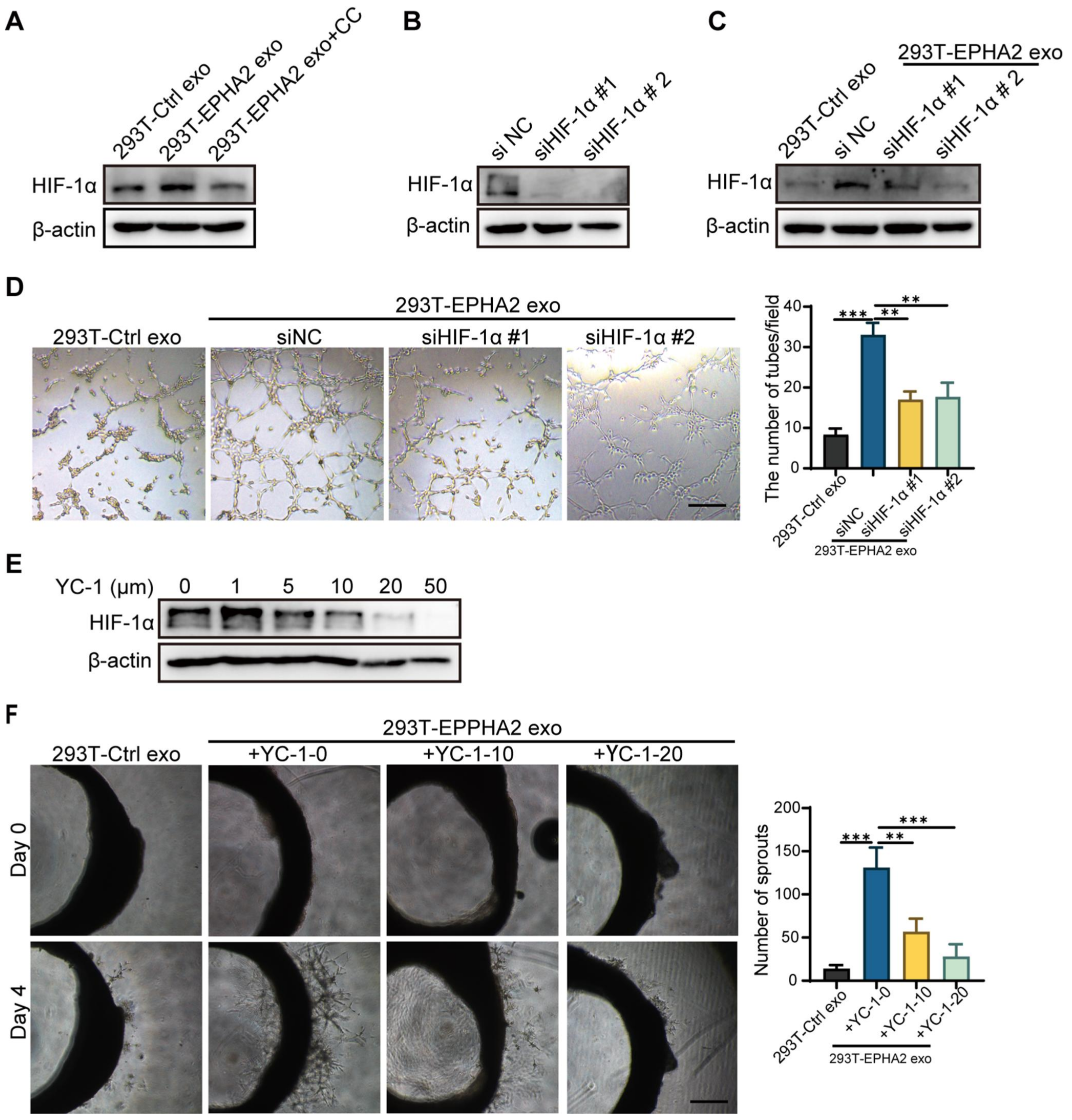


**Figure S4**

**A** EPHA2-rich exosomes increased HIF-1α protein levels in endothelial cells whereas Compound C inhibited the upregulation of HIF-1α by EPHA2-rich exosomes. **B** HIF-1α expression was silenced in endothelial cells. **C** EPHA2-rich exosomes failed to increase the HIF-1α protein levels in HIF-1α-KD cells. **D** EPHA2-rich exosomes failed to promote the tube-forming ability of HIF-1α-KD HUVECs. **E** YC-1 can inhibit the protein expression level of HIF-1α in endothelial cells. **F** YC-1 reduced the ability of microvessel growth in EPHA2-rich exosome-treated rat arterial rings.

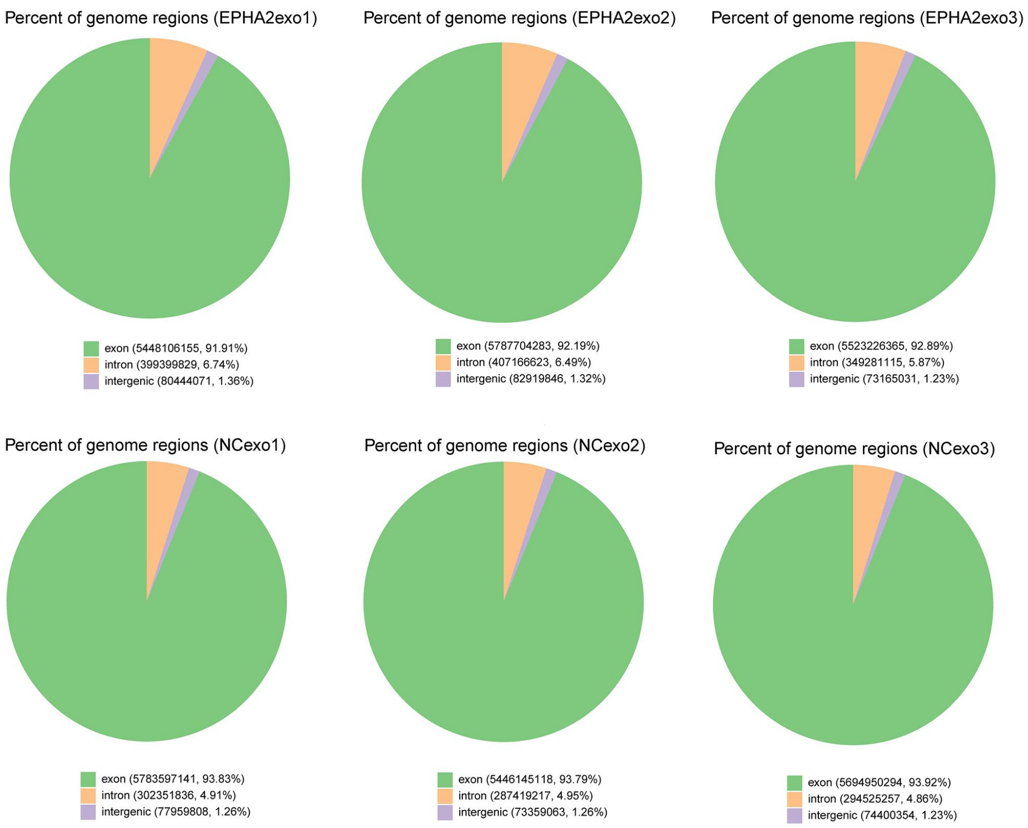


**Figure S5 The percentage of reads that mapped to the mRNA**

The percentage of reads that mapped to the mRNA. The mapping information of each technical replicate was shown as pie plot.

## Supplementary Tables

**Table S1. The shRNA sequences used in this study**

| Name | Sequence |
|---|---|
| shEPHA2 #1 | 5′-CTATTCTGTCAGTGTTAAA-3′ |
| shEPHA2 #2 | 5′-GATAAGTTTCTATTCTGTCAG-3′ |
| shEphrin-A1 #1 | 5′-AGAGGTGCGG GTTCTACATAG-3′ |
| shEphrin-A1 #2 | 5′-GTCTTCTGGAACAGTTCAAAT-3′ |

**Table S2. Primers used in this study**

| Name | Primers |
|---|---|
| mCherry-F | ACCTCCATAGAAGATTCTAGAGCCACCATGGTGAGCAAGGGCGAGGA |
| mCherry-R | TTCGAATTCGCTAGCTCTAGATTACTTGTACAGCTCGTCCATGCC |
| EPHA2-F | ACCTCCATAGAAGATTCTAGAGCCACCATGGAGCTCCAGG |
| EPHA2-R | TTCGAATTCGCTAGCTCTAGATTACTTGTACAGCTCGTCCATGCC |
| EPHA2-ΔRBD-F | ACCTCCATAGAAGATTCTAGAGCCACCATGGGCCTGGC |
| EPHA2-ΔRBD-R | TTCGAATTCGCTAGCTCTAGATTACTTGTACAGCTCGTCCATGCC |
| EPHA2-S897A-F | ACCTCCATAGAAGATTCTAGAGCCACCATGGAGCTCCAGG |
| EPHA2-S897A-R | TTCGAATTCGCTAGCTCTAGACTTACTTGTACAGCTCGTCCATGCC |
| EPHA2-D739N-F1 | ACCTCCATAGAAGATTCTAGAGCCACCATGGAGCTCCAGG |
| EPHA2-D739N-R1 | GGCAGCCAGATTACGGTGCACATAGTTCATGTTGGCC |
| EPHA2-D739N-F2 | GTGCACCGTAATCTGGCTGCCCGCAACATCCTCGTCAA |
| EPHA2-D739N-R2 | TTCGAATTCGCTAGCTCTAGACTTACTTGTACAGCTCGTCCATGCC |
| EPHA2-D739N-F3 | CTCCCCAGCACGAGCGGCTCGGAGGGGGTGCCCTTCCGCAC |
| EPHA2-D739N-R3 | AGCCGCTCGTGCTGGGGAGCCGGATAGACACGC |

**Table S3. Summary of RNA-Seq data quality**

| Sample | Raw reads | Clean reads | Clean bases | Error rate | Q20 | GC % |
|---|---|---|---|---|---|---|
| EphA2exo1 | 45324432 | 42280544 | 6.34G | 0.03 | 97.59 | 49.37 |
| EphA2exo2 | 45356986 | 44599476 | 6.69G | 0.03 | 97.85 | 48.95 |
| EphA2exo3 | 45116568 | 42306052 | 6.35G | 0.03 | 97.5 | 49.46 |
| NCexo1 | 45727976 | 44177416 | 6.63G | 0.03 | 97.72 | 50.57 |
| NCexo2 | 43439160 | 41605440 | 6.24G | 0.03 | 97.44 | 50.4 |
| NCexo3 | 46196778 | 43249020 | 6.49G | 0.03 | 97.51 | 50.31 |

## Table S4. Statistics of comparisons between samples and reference genome

| Exported data | | | | | | | | | | | |
|---|---|---|---|---|---|---|---|---|---|---|---|
| **sample** | **totalreads** | **total_map** | **unique_map** | **multi_map** | **read1_map** | **read2_map** | **positive_map** | **negative_map** | **splice_map** | **unsplice_map** | **proper_map** |
| Eph A2exo1 | 42280544 | 39617991(93.7%) | 38454350(90.95%) | 1163641(2.75%) | 19259293(45.55%) | 19195057(45.4%) | 19206818(45.43%) | 19247532(45.52%) | 17625455(41.69%) | 20828895(49.26%) | 37304646(88.23%) |
| Eph A2exo2 | 44599476 | 41929106(94.01%) | 40716734(91.29%) | 1212372(2.72%) | 20440495(45.83%) | 20276239(45.46%) | 20341504(45.61%) | 20375230(45.68%) | 18571138(41.64%) | 22145596(49.65%) | 39770516(89.17%) |
| Eph A2exo3 | 42306052 | 39732287(93.92%) | 38579694(91.19%) | 1152593(2.72%) | 19329588(45.69%) | 19250106(45.5%) | 19272114(45.55%) | 19307580(45.64%) | 18213613(43.05%) | 20366081(48.14%) | 37464970(88.56%) |
| NCexo1 | 44177416 | 41187863(93.23%) | 40058822(90.68%) | 1129041(2.56%) | 20091787(45.48%) | 19967035(45.2%) | 20010291(45.3%) | 20048531(45.38%) | 18815970(42.59%) | 21242852(48.09%) | 39001004(88.28%) |
| NCexo2 | 41605440 | 38796898(93.25%) | 37738365(90.71%) | 1058533(2.54%) | 18922335(45.48%) | 18816030(45.22%) | 18849861(45.31%) | 18888504(45.4%) | 17783513(42.74%) | 19954852(47.96%) | 36715388(88.25%) |
| NCexo3 | 43249020 | 40523961(93.7%) | 39372162(91.04%) | 1151799(2.66%) | 19718881(45.59%) | 19653281(45.44%) | 19667003(45.47%) | 19705159(45.56%) | 18732394(43.31%) | 20639768(47.72%) | 38235666(88.41%) |
